# Editorial: Integrating Time & Number: From Neural Bases to Behavioral Processes Through Development and Disease

**DOI:** 10.3389/fnhum.2020.00129

**Published:** 2020-04-09

**Authors:** Fuat Balcı, Metehan Çiçek, Karin Kucian, Trevor B. Penney

**Affiliations:** ^1^Department of Psychology, College of Social Sciences and Humanities, Koç University, Sariyer, Turkey; ^2^Department of Physiology, School of Medicine, Ankara University, Ankara, Turkey; ^3^Department of Interdisciplinary Neuroscience, Ankara University, Ankara, Turkey; ^4^Brain Research Center, Ankara University, Ankara, Turkey; ^5^Center for MR Research, University Children's Hospital Zurich, Zurich, Switzerland; ^6^Children's Research Center, University Children's Hospital Zurich, Zurich, Switzerland; ^7^Neuroscience Center Zurich, Zurich, Switzerland; ^8^Department of Psychology, The Chinese University of Hong Kong, Shatin, China

**Keywords:** interval timing, numerosity, mental magnitudes, psychophysics, dyscalculia, dyslexia, schizophrenia

Although crucial overlap exists in time and number processing, a thorough explanation of how neurons integrate time and number is lacking. Moreover, researchers have demonstrated the clinical relevance of timing and counting in disorders directly relevant to these conditions (e.g., dyscalculia, autism spectrum disorder) or others associated with dopaminergic dysfunction (e.g., Parkinson's Disease, Schizophrenia, ADHD).

The present Research Topic comprises a collection of papers evaluating the neural mechanisms of time and number perception, as well as the diseases which disturb these functions. A wide range of studies, from behavioral, psychophysical, neuroimaging, clinical, to theoretical, and from childhood to adulthood, as well as from typical to atypical processing, provide broad insights into time and number perception in the present Research Topic.

## Time Perception Studies

Apaydın et al. used event-related fMRI to investigate the interaction of time perception with reward prospects. Participants performed a time perception task in which they judged the velocity of an occluded moving object in “reward” vs. “no-reward” sessions. Findings showed a right-lateralized fronto-parietal activity during timing. Interaction of time and reward showed prefrontal and caudate nucleus activity, which suggests that reward and timing systems of the brain might be integrated in prefrontal-striatal circuitry.

Coull et al. used a temporal bisection task with lateralized arrow direction (to the left/right side) and/or lateralized stimulus (on the left/right side) presentation with children and adults to assess the beginning of mental timeline acquisition. Their findings suggest that while spatial position manipulated in a symbolic way influenced the duration judgments of 8 and 10 year olds, for 5–6 year olds only lateralized stimulus presentation affected timing. In another study, Karşılar et al. tested the effect of observed motion on time perception. They used animations of a walking stick-figure with different movement speeds and directions. Their findings suggest that (irrespective of the direction of motion) time dilates while observing faster motion and it contracts while observing slower motion.

Maaß and van Rijn aimed to assess whether clock variability could be reliably measured with an easy 1-s production task, which could then be applied in clinical and/or developmental studies. They suggest that the observed variability adheres to the scalar property and predicts temporal performance observed in a reproduction task. Zeki and Balcı presented a model of time cells with sequentially firing neurons and noisy conductances that exhibit a simple neural wave activity and showed that this simplified model of time cells can account for the prominent properties of the timing behavior including the scalar property.

## Studies Evaluating Time and Number Perception Together

Hamamouche et al. tested the effect of cognitive load on temporal and numerical processing in an attempt to determine whether the common magnitude system was supported. Participants performed numerical and temporal judgements under the distraction of a verbal working memory task. Results were inconsistent with the common magnitude account. On the other hand, Light et al. trained mice to perform Mechner's counting task in which animals had to press one lever for a required number of times before claiming the reward by pressing a second lever. The results indicate that mice used both counting and timing strategies to complete the task.

Agostino et al. evaluated the estimation of subjective magnitude of long-range time intervals and tested the effect of numerals in duration judgements. The results showed that people map the magnitude of stimuli presented as an abstract number and number + time-unit in a similar way, while time interval magnitudes without the use of numerals (e.g., personal events) are treated differently.

## Time and Number Processing Deficits in Disease States

McCaskey et al. investigated the numerical abilities of typically developing children and those with developmental dyscalculia (DD) by using neuropsychological tests and fMRI scanning during a numerical order paradigm. The results showed that children with DD showed persistent deficits in number processing and arithmetical skills, but they showed an improvement and their brain imaging results revealed an increase in frontal and parietal brain activation over time.

Di Filippo and Zoocolatti evaluated 5 children with dyslexia, 16 with dyscalculia, 7 with a “mixed pattern,” and 49 control children in terms of reading and numerical skills. They showed that the deficit of children (with dyscalculia and mixed pattern) on numerical tasks could be described by a single global factor.

Snowden and Buhusi reviewed the neuroimaging literature related to the neural correlates of interval timing problems in schizophrenia. Based on previous work the authors suggest that a disrupted cortico-striatal-thalamo-cortical network may be responsible for timing deficits observed in schizophrenia. Furthermore, Xiong et al. investigated electrophysiological differences in patients with first-episode schizophrenia and chronic schizophrenia. Their findings suggest that duration and frequency mismatch negativity and evoked theta power differences may be used to detect schizophrenia at early stages and are related to poor cognitive functioning in schizophrenic patients.

## Author Contributions

All authors listed have made a substantial, direct and intellectual contribution to the work, and approved it for publication.

### Conflict of Interest

The authors declare that the research was conducted in the absence of any commercial or financial relationships that could be construed as a potential conflict of interest. The handling Editor declared a shared affiliation, though no other collaboration, with one of the authors KK.

